# A Non-Isothermal Moving-Boundary Model for Continuous and Intermittent Drying of Pears

**DOI:** 10.3390/foods9111577

**Published:** 2020-10-30

**Authors:** Alessandra Adrover, Claudia Venditti, Antonio Brasiello

**Affiliations:** 1 Dipartimento di Ingegneria Chimica, Materiali e Ambiente, Sapienza Università di Roma, via Eudossiana 18, 00184 Roma, Italy; claudia.venditti@uniroma1.it (C.V.); antonio.brasiello@uniroma1.it (A.B.); 2 INSTM Consorzio Interuniversitario Nazionale per la Scienza e Tecnologia dei Materiali, Via G. Giusti 9, 50121 Firenze, Italy

**Keywords:** intermittent dehydration, shrinkage, moving-boundary model, non-isothermal drying

## Abstract

A non-isothermal moving-boundary model for food dehydration, accounting for shrinkage and thermal effects, is proposed and applied to the analysis of intermittent dehydration in which air temperature, relative humidity, and velocity vary cyclically in time. The convection-diffusion heat transport equation, accounting for heat transfer, water evaporation, and shrinkage at the sample surface, is coupled to the convection-diffusion water transport equation. Volume shrinkage is not superimposed but predicted by the model through the introduction of a point-wise shrinkage velocity. Experimental dehydration curves, in continuous and intermittent conditions, are accurately predicted by the model with an effective water diffusivity $D_{\mathrm{eff}}\left(T\right)$ that depends exclusively on the local temperature. The non-isothermal model is successfully applied to the large set of experimental data of continuous and intermittent drying of *Rocha* pears.

## 1. Introduction

Food process engineering represents one of the research fields that could benefit most from theoretical/computational support, that is the accurate modeling of all the complex heat and mass transport phenomena involved in many processes of interest to the food industry.

Natural and convective drying, for food production and preservation, is undoubtedly one of the most investigated processes [1]. It involves heat and mass transport in a shrinking food sample [2]. Shrinkage is a major phenomenon connected to drying [3] since it influences consumer quality perception, costs for transportation and storage. Mathematical modeling of drying is a useful tool for optimizing the process and designing the dryer.

Most modeling approaches for the description of convective dehydration neglect thermal phenomena and assume that the temperature is uniform within the sample and equal to the temperature of the air in the climatic chamber. For a detailed review and classification of theoretical models for the convective drying of fruits see the recent review by Castro et al. [4].

Thermal effects cannot be overlooked in the analysis and modeling of intermittent dehydration [5,6,7,8,9,10,11] in which the air properties change during the process. Intermittent dehydration has the technical advantage of increasing the dehydration capacity per unit energy consumption, improving product quality and reducing color degradation due to non-enzymatic browning. For this reason, intermittent drying is widely applied for dehydration of heat-sensitive bioproducts (see [8] and references therein).

A very interesting paper by Silva et al. [11] recently analyzed an intermittent drying process of whole *Rocha* pears in which air temperature, relative humidity, and velocity vary cyclically in a climatic chamber specifically designed to reproduce the traditional sun-drying of the Portuguese *Sao Bartolomeu* pears. Pears are characterized by a high initial moisture content and exhibit a large *ideal* shrinkage [12], meaning that volume reduction is equal to the volume of water removed during drying.

This article stems from the idea of verifying the predictive capabilities of the moving-boundary dehydration model, recently proposed by Adrover et al. [13,14], by analyzing the large set of experimental data of intermittent drying of *Rocha* pears reported by Silva et al. [11].

This isothermal moving-boundary model has been already successfully applied to describe the continuous dehydration kinetics and shrinkage of different food materials and sample shapes, e.g., eggplant cylindrical [13] and discoidal samples [15], chayote slices [16], potatoes sticks [14] and square slices [13], ellipsoidal cocoa beans [17].

The aim of this work is to improve the moving-boundary model to account for thermal effects. To this end, a convection-diffusion heat transport equation, accounting for sample shrinkage, heat transfer and water evaporation at the sample surface, is added to the convection-diffusion water transport equation. Like in the isothermal moving-boundary model, volume shrinkage is not superimposed but predicted by the model via the introduction of the pointwise shrinkage velocity that depends on the local volumetric water flux. The predictive capabilities of the model have been checked onto the experimental intermittent deydration curves of *Rocha* pears, performed in a programmable climatic chamber, and simulating the cyclic repetition of the three different stages characterizing the traditional solar drying. Numerical results clearly show that, if the spatio-temporal evolution of the temperature field is properly accounted for, the experimental dehydration curves, in continuous and intermittent conditions, can be accurately predicted by the moving-boundary model with an effective water diffusivity $D_{\mathrm{eff}}\left(T\right)$ that depends exclusively on the local temperature.

The article is organized as follows. Section 2 reviews the morphological and geometrical parameters of spherical pears subjected to dehydration and briefly describe the operating conditions for continuous and intermittent drying, as reported by [11]. Section 3 reviews the basic equations and boundary conditions of the isothermal moving-boundary model and presents its corresponding non-isothermal formulation. Section 4 focuses on continuous dehydration experiments and shows that the isothermal model does not provide satisfactory results. This is because the temperature of the whole pear T(r,t) cannot be approximated with the air temperature $T_{\infty }$, even in the simpler continuous dehydration process. However, the isothermal approach allows estimating the effective water diffusivity $D_{\mathrm{eff}}\left(T\right)$ from the asymptotic exponential behaviour of the two continuous dehydration curves at $T_{\infty }=40$ ${}^{\circ }$C and 50 ${}^{\circ }$C, as discussed in Section 4.2 and in Appendix B. The water diffusivity $D_{\mathrm{eff}}\left(T\right)$ is the only unknown parameter that enters the non-isothermal model. All the other parameters have been estimated from independent measurements (e.g., desorption isotherms) or from reliable correlations (e.g., heat and mass transfer coefficients $h_{T}$ and $h_{m}$), as discussed in Appendix A. The non-isothermal model is successfully applied, in a fully predictive way, to describe the continuous dehydration experiments in Section 4.3, and the intermittent dehydration tests in Section 5. The influence of air velocity on the dehydration time and the effectiveness of the pause stages on the reduction of moisture internal gradients are also addressed in Section 5.

## 2. Continuous and Intermittent Drying of *Rocha* Pears

We analyze experimental data of continuous and intermittent drying of pears of *Rocha* variety reported in [11] and summarized in Table 1.

**Figure 1:**
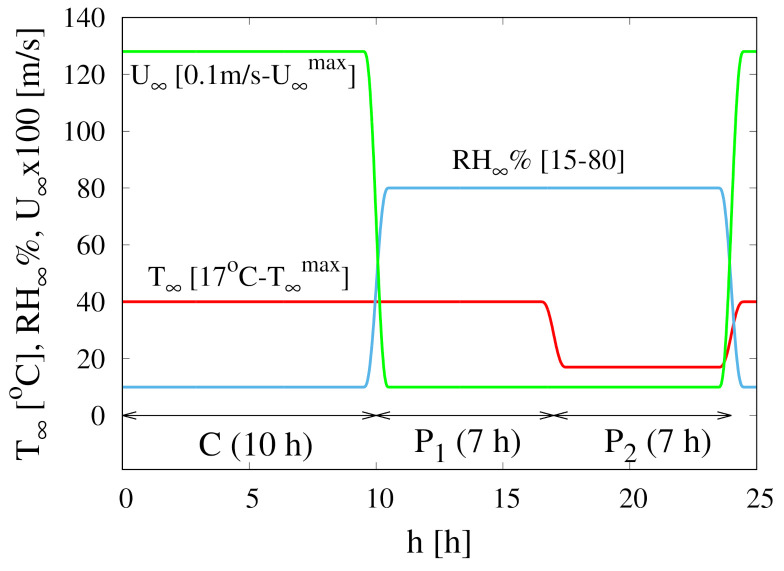
Temporal evolution of air temperature $T_{\infty }$, Relative Humidity $RH_{\infty }$ and velocity $U_{\infty }$ during the three distinct stages of a single cycle for intermittent dehydration, namely a convective high-temperature drying period (C), a high-temperature humid pause (P${}_{1}$) and a low-temperature humid pause (P${}_{2}$). $U_{\infty }^{\max}=1.28$ or 2.66 m/s. $T_{\infty }^{\max}=40$ ${}^{\circ }$C or 50 ${}^{\circ }$C.

Continuous and intermittent drying experiments are performed in a programmable climatic chamber simulating the cyclic repetition of the three different stages (see Figure 1) characterizing the traditional solar drying, namely

1. A first stage (10 h) of convective drying (C) with air velocity $U_{\infty }^{\max}=1.28$ or 2.66 m/s, high temperature $T_{\infty }^{\max}=40$ ${}^{\circ }$C or 50 ${}^{\circ }$C and low Relative Humidity RH=15%.
2. A second pause stage ($P_{1}$, 7 h), simulating the barreling stage, characterized by a high temperature $T_{\infty }=40$ ${}^{\circ }$C, 50 ${}^{\circ }$C, high Relative Humidity RH=80% and very low air velocity $U_{\infty }<0.2$ m/s.
3. A third pause stage ($P_{2}$, 7 h), simulating the night period, characterized by a low temperature $T_{\infty }=17$ ${}^{\circ }$C, high Relative Humidity RH=80% and very low air velocity $U_{\infty }<0.2$ m/s.

For details regarding the experimental setup see the original paper by Silva et al. [11].

**Figure 2:**
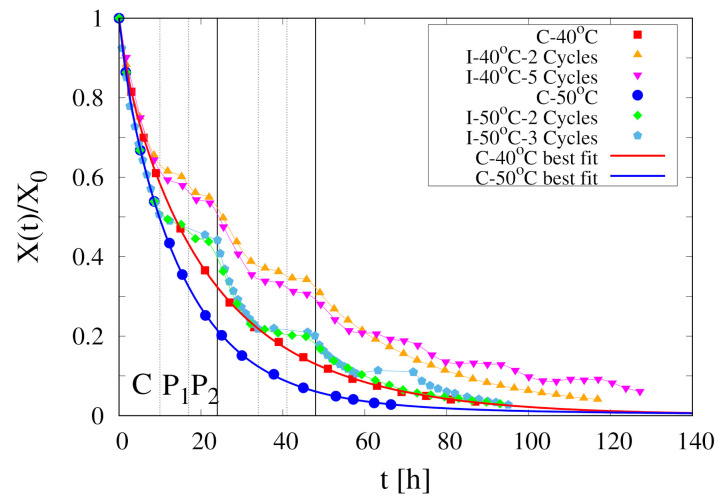
Collection of experimental data for continuous and intermittent drying of spherical pears at $T_{\infty }=40$ ${}^{\circ }$C, 50 ${}^{\circ }$C, $U_{\infty }=1.28$ m/s. Vertical lines highlight the different stages of the first two cycles for intermittent drying experiments. Continuous thick (red and blue) lines represent the best-fit curves, Equation (22), for the two continuous drying experiments *C*-40 ${}^{\circ }$C and *C*-50 ${}^{\circ }$C.

The collection of experimental data for the temporal evolution of the rescaled total moisture content $X\left(t\right)/X_{0}$ for continuous and intermittent drying of spherical pears at T=40 ${}^{\circ }$C, 50 ${}^{\circ }$C and $U_{\infty }=1.28$ m/s are reported in Figure 2 where vertical lines highlight the different stages of the first two cycles for intermittent drying experiments.

Details regarding the initial total moisture content $X_{0}$ and the initial sample diameter $d_{0}$ for each experiment are reported in Table 1.

## 3. Isothermal and Non-Isothermal Moving-Boundary Models

This section preliminary reviews the basic idea and the resulting equations of the moving-boundary model for food isothermal dehydration, developed in [13,14]. It subsequently extends the moving-boundary model to the non-isothermal case in which the sample temperature cannot be assumed constant (in time and/or space) and equal to the air temperature $T_{\infty }$ of the climatic chamber.

### 3.1. Isothermal Moving-Boundary Model

During the dehydration process, the sample volume V(t) and surface S(t) evolve in time due to sample shrinkage. The sample temperature is assumed constant in space and time and equal to the air temperature $T_{\infty }$.

The transport equation describing the space-time evolution of the pointwise water concentration $c_{w}\left(x,t\right)$ [g water/m${}^{3}$ product] inside the sample volume V(t) is an advection-diffusion equation accounting for the local shrinkage through the pointwise shrinkage velocity $v_{s}\left(x\right)$

(1) $$\frac{\partial c_{w}\left(x,t\right)}{\partial t}-\nabla \;\cdot \;\left(J_{d}+v_{s}\left(x\right)\;c_{w}\right)\;=\;=\nabla \;\cdot \;\left(D_{\mathrm{eff}}\;\nabla c_{w}-v_{s}\left(x\right)\;c_{w}\right)\;,\;x\in V\left(t\right)$$

where $J_{d}=-D_{\mathrm{eff}}\;\nabla c_{w}$ is the diffusive mass flux, controlled by the effective water diffusivity $D_{\mathrm{eff}}$, and $\left(v_{s}c_{w}\right)$ is a convective term arising from local shrinkage.

By enforcing the analogy between food dehydration and swelling of rubbery polymers (both processes are characterized by moving boundaries whose movement is controlled by water release or absorption [18,19,20,21].), the pointwise shrinkage velocity $v_{s}\left(x\right)$ is assumed proportional (and opposite in sign) to the diffusive volumetric flux $J_{d}\left(x\right)/\rho_{w}$ [m${}^{3}$ water/(s m${}^{2}$)]

(2) $$v_{s}\left(x\right)=-\alpha \left(c_{w}\right)\;J_{d}\left(x\right)/\rho_{w}=\alpha \left(c_{w}\right)\;D_{\mathrm{eff}}\;\nabla c_{w}/\rho_{w}\;,$$

where $\alpha (c_{w})$ is a shrinkage proportionality factor, depending on the pointwise water concentration.

The shrinkage factor $\alpha (c_{w})$ is the fingerprint of the specific food material under investigation. The simplest case is that of a constant shrinkage factor, i.e., $\alpha \left(c_{w}\right)=\alpha_{0}$. $\alpha_{0}=0$ represents the case of a rigid solid (no shrinkage). $\alpha_{0}=1$ represents the case of *ideal* shrinkage, in which volume reduction corresponds exactly to the volume of water flowing outside the sample. Values of $\alpha_{0}$ less or greater than unity imply volume reduction less or greater than the corresponding water volume flow [14,15].

The effective water diffusivity $D_{\mathrm{eff}}$ can be assumed constant in space or, in a more refined approach, it can be expressed as an increasing exponential function of the water volume fraction ϕ [18,19]

(3) $$D_{\mathrm{eff}}\left(\phi ,T\right)=D_{\phi_{0}}\left(T\right)\exp\left(-\beta \;\frac{\phi_{0}-\phi }{\phi_{0}-\phi_{\infty }}\right)\;,\;\beta \geq 0\;,\;\phi =c_{w}/\rho_{w}$$

In Equation (3) $\phi_{0}$ is the initial water volume fraction, $D_{\phi_{0}}$ and $D_{\infty }=D_{\phi_{0}}\exp\left(-\beta \right)$ are the effective diffusivities at the beginning and at the end of the drying process, respectively.

The shrinkage velocity $v_{s}\left(x_{b}\right)$, at every point $x_{b}$ on the sample boundary S(t), controls the temporal evolution of the sample boundary S(t) according to the following equation

(4) $$\frac{dx_{b}}{dt}=v_{s}{|}_{x_{b}}=\frac{\alpha (c_{w})}{\rho_{w}}D_{\mathrm{eff}}\;\nabla c_{w}{|}_{x_{b}}\;,\;x_{b}\in S\left(t\right)\;.$$

The two transport equations Equations (1) and (4) are linked together and must be solved simultaneously by further enforcing the following mixed boundary condition, also referred to as Robin or “evaporative” or third order boundary condition [10]

(5) $$-D_{\mathrm{eff}}\;\nabla c_{w}\cdot n{|}_{x_{b}}=h_{m}\;M_{w}\left({C|}_{x_{b}}-C_{\infty }\right)\;=\;=h_{m}\;M_{w}\frac{p_{v}\left(T_{\infty }\right)}{R_{g}T_{\infty }}\left(RH_{b}-RH_{\infty }\right)$$

where n is the outward-pointing normal unit vector, $M_{w}=18$ [g/mol] the water molecular weight, $h_{m}$ [m/s] the mass transfer coefficient, $p_{v}\left(T_{\infty }\right)$ the saturated vapor pressure at the air temperature $T_{\infty }$. *C* [mol/m${}^{3}$] is the water (vapor) concentration in air and can be further expressed in terms of the Relative Humidity RH, at the air/sample interface $RH{|}_{x_{b}}$ and in the climatic chamber $RH_{\infty }$, by adopting the ideal gas law

(6) $$C=\frac{p}{R_{g}T}=\frac{p_{v}\left(T\right)}{R_{g}T}\frac{p}{p_{v}\left(T\right)}=\frac{p_{v}\left(T\right)}{R_{g}T}\;RH$$

The Relative Humidity $RH_{b}=RH{|}_{x_{b}}$ at the air/sample interface, depends on the local water concentration $c_{w}\left(x_{b},t\right)$ and on the temperature $T_{\infty }$ and must be evaluated from the Desorption Isotherm (DI) at $T=T_{\infty }$

(7) $$RH_{b}=RH{|}_{x_{b}}=DI\left(c_{w}\left(x_{b},t\right),T_{\infty }\right)$$

The mass transfer coefficient $h_{m}$ can be evaluated from well-known correlation functions for the Sherwood number Sh, specific for the sample geometry under investigation (sphere, cylinder, ellipsoid, slab) with all the physical parameters of the humid air evaluated at $T_{\infty }$ (see Appendix A).

### 3.2. Non-Isothermal Moving-Boundary Model

In the non-isothermal approach a partial differential equation describing the spatio-temporal evolution of the temperature T(x,t) in the shrinking sample is coupled to the mass transport equations Equations (1), (4) and (5). Equations (1), (4) and (5) need to be slightly modified to take into account that the internal temperature T(x,t) and the boundary temperature $T_{b}=T\left(x_{b},t\right)$ are different from the air temperature $T_{\infty }$ in the climatic chamber.

The non-isothermal approach requires the simultaneous solution of the two advection-diffusion partial differential equations for $c_{w}\left(x,t\right)$ and T(x,t)

(8) $$\begin{array}{ccc} \frac{\partial c_{w}\left(x,t\right)}{\partial t} & = & \nabla \;\cdot \;\left(D_{\mathrm{eff}}\left(T\right)\;\nabla c_{w}-v_{s}\left(x\right)\;c_{w}\right)\;,\; \end{array}$$

(9) $$\begin{array}{ccc} \frac{\partial \left(\rho^{p}C_{p}^{p}T\left(x,t\right)\right)}{\partial t} & = & \nabla \;\cdot \;\left(k^{p}\;\nabla T-v_{s}\left(x\right)\;\rho^{p}C_{p}^{p}T\right)\;,\; \end{array}$$

(10) $$\begin{array}{ccc} v_{s}\left(x\right) & = & \alpha \left(c_{w}\right)D_{\mathrm{eff}}\left(T\right)\nabla c_{w}/\rho_{w} \end{array}$$

coupled with the equation for the temporal evolution of sample boundary S(t)

(11) $$\frac{dx_{b}}{dt}=v_{s}{|}_{x_{b}}=\frac{\alpha (c_{w})}{\rho_{w}}D_{\mathrm{eff}}\left(T_{b}\right)\;\nabla c_{w}{|}_{x_{b}}\;,\;x_{b}\in S\left(t\right)\;.$$

and with the boundary conditions

(12) $$-D_{\mathrm{eff}}\left(T_{b}\right)\;\nabla c_{w}\cdot n{|}_{x_{b}}=h_{m}\left(T_{\mathrm{av}}\right)\;M_{w}\left(\frac{p_{v}\left(T_{b}\right)}{R_{g}T_{b}}RH_{b}-\frac{p_{v}\left(T_{\infty }\right)}{R_{g}T_{\infty }}RH_{\infty }\right)$$

(13) $$-k^{p}\;\nabla T\cdot n{|}_{x_{b}}=h_{T}\left(T_{\mathrm{av}}\right)\left(T_{b}-T_{\infty }\right)-\lambda_{v}\left(T_{b}\right)D_{\mathrm{eff}}\left(T_{b}\right)\nabla c_{w}\cdot n{|}_{x_{b}}$$

The boundary condition Equation (13) takes into account both the heat transfer resistance and the heat subtracted for water evaporation at the air/sample interface [22,23,24,25,26], $\lambda_{v}$ being the heat of water evaporation, evaluated at $T_{b}$.

In this non-isothermal case, the Relative Humidity $RH_{b}$ at the air/sample interface depends on the local water concentration $c_{w}\left(x_{b},t\right)$ and on the boundary temperature $T_{b}\neq T_{\infty }$.

The heat and mass transfer coefficients $h_{T}$ and $h_{m}$ can be evaluated from well-known correlation functions for the Sherwood Sh and Nusselt Nu numbers, specific for the sample geometry under investigation, with all the physical parameters of the humid air evaluated at the average film temperature $T_{\mathrm{av}}=\left(T_{b}+T_{\infty }\right)/2$ (see Appendix A).

All the physical parameters of the food product, namely the product density $\rho^{p}$, the specific heat capacity $C_{p}^{p}$ and the thermal conductivity $k^{p}$, are functions of the local water concentration $c_{w}$ (see Appendix A).

In the present formulation it has been assumed that the effective water diffusivity is solely a function of the temperature and independent of the local water concentration, i.e., β=0 in Equation (3).

The heat transport equation Equation (9) is coupled to the mass transport equation Equation (8) not only through the boundary condition Equation (13) but also through the shrinkage-convective term $\left(v_{s}\left(x\right)\;\rho^{p}C_{p}^{p}T\right)$ that contributes to flatten the temperature profile inside the sample.

If the thermal diffusivity $D_{T}^{p}=k^{p}/\left(\rho^{p}C_{p}^{p}\right)$ of the food material is significantly larger than water diffusivity $D_{\mathrm{eff}}$, we can assume that the temperature is uniform inside the sample and equal to the boundary temperature, i.e., $T\left(x,t\right)=T_{b}\left(t\right)$. Consequently, the partial differential equation Equation (9) for T(x,t) can be replaced with the ordinary differential equation describing the temporal evolution of the boundary temperature $T_{b}\left(t\right)$

(14) $$\frac{d}{dt}\left(T_{b}\int_{V(t)}\left(\rho^{p}C_{p}^{p}\right)\;dx\right)=\int_{S(t)}\left(-h_{T}\left(T_{\mathrm{av}}\right)\left(T_{b}-T_{\infty }\right)+\lambda_{v}\left(T_{b}\right)D_{\mathrm{eff}}\left(T_{b}\right)\nabla c_{w}\cdot n{|}_{x_{b}}\right)dS$$

while the water transport equations Equations (8) and (10) remain unchanged except for the fact that $D_{\mathrm{eff}}\left(T\right)$ must be replaced with $D_{\mathrm{eff}}\left(T_{b}\right)$.

### 3.3. Numerical Issues

PDE equations and boundary conditions describing the one-dimensional shrinkage dynamics and sample dehydration have been numerically solved using finite elements method (FEM) in Comsol Multiphysics 3.5. The convection–diffusion package coupled with ALE (Arbitrary Lagrangian Eulerian) moving mesh has been adopted with Free Displacement induced by boundary velocity conditions. Lagrangian quadratic elements have been chosen. The linear solver adopted is UMFPACK, with relative tolerance $10^{-4}$ and absolute tolerance $10^{-7}$. The Time Stepping Method adopted is BDF with a Strict policy for time steps taken by the solver in order to have a good resolution (in time) of step changes in boundary conditions (air temperature, relative humidity and velocity). The number of finite elements set to $10^{4}$ with a non-uniform mesh. Smaller elements have been located close to the moving boundary r=R(t) in order to accurately compute concentration and temperature gradients, controlling the velocity of the moving front.

## 4. Modeling of Continuous Drying Experiments

### 4.1. The Isothermal Approach

We preliminary adopt the isothermal approach to model the two continuous drying experiments on spherical pears at T=40 ${}^{\circ }$C, 50 ${}^{\circ }$C (experimental data shown in Figure 2).

It is assumed that the spherical shape of the sample is not altered by the dehydration process. The sample geometry is uniquely characterized by its radius R(t) evolving in time from its initial value $R_{0}$ towards its asymptotic value $R_{\infty }$.

According to experimental shrinkage data reported by Silva et al. [11] (subsequently shown in Figure 7B), it is assumed *ideal* shrinkage, i.e., that volume reduction equals, at each time instant, the volume of water released by the sample

(15) $$\left(1-\frac{V(t)}{V_{0}}\right)=\phi_{0}\;\left(1-\frac{X(t)}{X_{0}}\right)$$

where $\phi_{0}$ is the initial uniform water volume fraction. This macroscopic observation finds its microscopic counterpart in the assumption of a constant and unitary shrinkage coefficient $\alpha \left(c_{w}\right)=\alpha_{0}=1$. It must be pointed out that the assumption $\alpha \left(c_{w}\right)=\alpha_{0}$ does not imply that the shrinkage velocity $v_{s}\left(x\right)$ is constant, nor in time or space, but rather that, according to Equation (2), the shrinkage velocity is directly proportional to the local concentration gradient. Therefore, the shrinkage velocity asymptotically tends to zero, at each point in the sample, when the water concentration gradient goes to zero everywhere in the system, i.e., equilibrium conditions are reached.

The water transport equation and boundary conditions, Equations (1), (2) and (4), rewritten in spherical coordinates and in terms of the water volume fraction $\phi \left(r,t\right)=c_{w}\left(r,t\right)/\rho_{w}$, read as

(16) $$\frac{\partial \phi (r,t)}{\partial t}=\frac{1}{r^{2}}\frac{\partial }{\partial r}\left(r^{2}\left(D_{\mathrm{eff}}\;\frac{\partial \phi }{\partial r}-v_{s}\left(r\right)\;\phi \right)\right)\;,\;r\in \left(0,R\left(t\right)\right)$$

(17) $$v_{s}\left(r\right)=D_{\mathrm{eff}}\frac{\partial \phi }{\partial r}\;,\;\frac{dR(t)}{dt}=v_{s}\left(R\left(t\right)\right)=D_{\mathrm{eff}}\frac{\partial \phi }{\partial r}{|}_{R(t)}$$

(18) $$\frac{\partial \phi }{\partial r}{|}_{r=0}=0\;,\;-D_{\mathrm{eff}}\frac{\partial \phi }{\partial r}{|}_{R(t)}=h_{m}\frac{M_{w}}{\rho_{w}}\frac{p_{v}\left(T_{\infty }\right)}{R_{g}T_{\infty }}\left(RH_{b}-RH_{\infty }\right)$$

The Relative Humidity at the air/sample interface $RH_{b}=RH{|}_{R(t)}=RH\left(\phi_{b},T_{\infty }\right)$ is evaluated from desorption isotherms for *Rocha* pears reported by [27] and best fitted with the Henderson model, as discussed in detail in Appendix A. The correlation function adopted for the estimate of the mass transfer coefficient $h_{m}$, evaluated at the air temperature $T_{\infty }$, is also reported in Appendix A.

**Figure 3:**
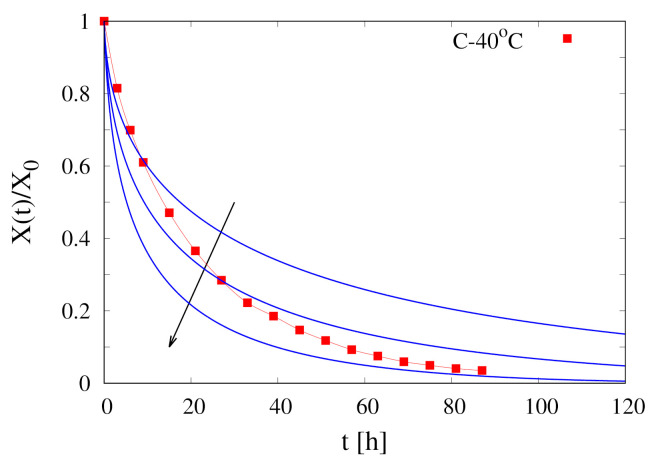
Continuous dehydration data $X\left(t\right)/X_{0}$ vs. time at $T_{\infty }=40$ ${}^{\circ }$C. Comparison between experimental data (points) and isothermal model predictions (continuous lines) with $D_{\mathrm{eff}}=5\times 10^{-11},1\times 10^{-10},2\times 10^{-10}$ m${}^{2}$/s. Arrow indicates increasing values of $D_{\mathrm{eff}}$.

Figure 3 shows the comparison between experimental data for $X\left(t\right)/X_{0}$ vs. time at $T_{\infty }=40$ ${}^{\circ }$C and isothermal model predictions with $D_{\mathrm{eff}}=5\times 10^{-11},1\times 10^{-10},2\times 10^{-10}$ m${}^{2}$/s. It can be readily observed that an isothermal model with a constant water diffusivity $D_{\mathrm{eff}}$ is not able to capture the salient features of the experimental dehydration curve. The lower value of $D_{\mathrm{eff}}$ can well approximate only the initial behaviour of the dehydration curve, while higher values of $D_{\mathrm{eff}}$ better describe the asymptotic behaviour. Even worse results would be obtained by using a diffusivity dependent on the volumetric water fraction Equation (3).

In order to understand this result, some observations must be done

1. By direct comparison between experimental continuous dehydration curves at $T_{\infty }=40$ ${}^{\circ }$C and $T_{\infty }=50$ ${}^{\circ }$C (see Figure 2) it can be observed that $D_{\mathrm{eff}}$ is highly sensitive to air temperature $T_{\infty }$
2. The initial temperature of the sample in all the experiments is T(r,0)≃15 ${}^{\circ }$C, well below the operating temperature $T_{\infty }=40$ ${}^{\circ }$C, 50 ${}^{\circ }$C
3. The initial moisture content $X_{0}$ of *Rocha* pears is high, order of 5–6 kg water/kg dry solid, that implies an initial water weight fraction $x_{w}\left(0\right)≃0.84$. To make a rough calculation, at the beginning of the drying process, the product density $\rho^{p}$, the specific heat capacity $C_{p}^{p}$ and the thermal conductivity $k^{p}$ can be reasonably approximated with that of water.
  A sphere of non evaporating water with diameter $d_{0}≃5.3$ cm requires about five hours to rise its temperature from 15 ${}^{\circ }$C to 40 ${}^{\circ }$C for a heat transfer coefficient $h_{T}≃20$ W/(m${}^{2}$ K).
4. Given the high dehydration rates, especially at the beginning of the drying process, most part of the heat flux supplied by forced convection $h_{T}\left(T_{\infty }-T_{b}\right)$ is used for water evaporation at the air/sample interface. Therefore, the time required to rise the sample temperature from 15 ${}^{\circ }$C to 40 ${}^{\circ }$C could reasonably increase from 5 to more than 30 h.

For all these reasons, it has to be expected that the water diffusivity $D_{\mathrm{eff}}$, at least in the first 30–40 h of the drying process, is changing in time due to its sensitivity to the time-dependent sample temperature. $D_{\mathrm{eff}}$ progressively increases from lower values, corresponding to lower sample temperatures, towards the asymptotic value $D_{\mathrm{eff}}\left(T_{\infty }\right)$ that settles when the sample temperature reaches the air temperature $T_{\infty }$. The adoption of a non-isothermal model is strictly necessary. The necessity to account for a temperature dependent diffusion coefficient, even in a continuous drying experiment, has been already pointed put by Srikiatden and Roberts [28,29] in dealing with convective hot air and isothermal drying of potatoes and carrots.

### 4.2. The Estimate of $D_{\mathrm{eff}}\left(T\right)$ from the Asymptotic Behavior of Dehydration-Rate Curves

Despite the fact that an isothermal model cannot be applied for an accurate description of the whole continuous dehydration curve, a simplified isothermal model can be used to estimate the effective diffusivity $D_{\mathrm{eff}}\left(T\right)$ from the asymptotic behaviour of the continuous dehydration curves.

On longer time scales, corresponding to lower values of the total moisture content $X\left(t\right)/X_{0}\leq 0.2$, we can reasonably assume that (1) the sample temperature is uniform and equal to $T_{\infty }$ and (2) the sample volume V(t) has reached its asymptotic value $V_{\infty }$ after almost complete shrinkage (see Figure 7B)

(19) $$\frac{V_{\infty }}{V_{0}}=1-\phi_{0}\left(1-\frac{X_{\infty }}{X_{0}}\right)≃0.1.$$

On longer time scales, the convective-shrinkage contribution to water transport becomes negligible and the dimensionless dehydration rate J(t)

(20) $$J\left(t\right)=-\frac{dX_{r}}{dt}=-\frac{d}{dt}\left(\frac{X-X_{\infty }}{X_{0}-X_{\infty }}\right)$$

becomes a linear function of the moisture ratio $X_{r}\left(t\right)$

(21) $$J\left(t\right)=D_{\mathrm{eff}}\left(T_{\infty }\right)\frac{\pi^{2}}{R_{0}^{2}}{\left(\frac{V_{\infty }}{V_{0}}\right)}^{-2/3}X_{r}\left(t\right)$$

The derivation of Equation (21) is reported in Appendix B.

**Figure 4:**
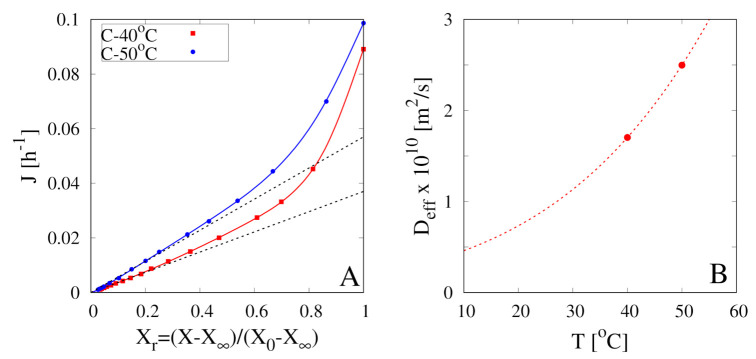
(**A**) Dehydration-rate curves *J* vs. $X_{r}$ for continuous dehydration tests at $T_{\infty }=40$ ${}^{\circ }$C, 50 ${}^{\circ }$C, $U_{\infty }=1.28$ m/s. Continuous lines represent the best-fit curves Equation (23). Black dashed lines highlight the asymptotic (large *t*, small $X_{r}$) linear behaviour, Equation (21). (**B**) Estimated effective water diffusivity $D_{\mathrm{eff}}$ at T=40 ${}^{\circ }$C, 50 ${}^{\circ }$C (filled points). The dashed line represents the Arrhenius behaviour, Equation (24).

Figure 4A shows the dehydration-rate curves *J* vs. $X_{r}$ for the two continuous dehydration experiments at $T_{\infty }=40$ ${}^{\circ }$C and 50 ${}^{\circ }$C. These curves are obtained from the best-fit of the corresponding dehydration curves $X\left(t\right)/X_{0}$ with the following function

(22) $$X\left(t\right)/X_{0}=a_{0}+a_{1}e^{-b_{1}t}+a_{2}e^{-b_{2}t}+\left(1-a_{0}-a_{1}-a_{2}\right)e^{-b_{3}t}$$

that satisfies the two constrains $X\left(0\right)/X_{0}=1$, $X_{\infty }/X_{0}=a_{0}$. The best-fit curves are shown in Figure 2 (red and blue thick lines). The dehydration rate J(t) can be subsequently evaluated as

(23) $$J\left(t\right)=\frac{1}{1-a_{0}}\left(a_{1}b_{1}e^{-b_{1}t}+a_{2}b_{2}e^{-b_{2}t}+\left(1-a_{0}-a_{1}-a_{2}\right)\;b_{3}e^{-b_{3}t}\right),$$

and plotted as a function of $X_{r}=\frac{\left(X\left(t\right)/X_{0}\right)-a_{0}}{1-a_{0}}$.

The experimental dehydration-rate curves, shown in Figure 4A, exhibit the expected asymptotic linear behaviour, Equation (21), valid for large *t* or equivalently for small $X_{r}$. From these dehydration-rate curves and Equation (21) the following values of water diffusivity $D_{\mathrm{eff}}(40$ ${}^{\circ }$C$)\;=\;1.703\;\times \;10^{-10}$ m${}^{2}$/s and $D_{\mathrm{eff}}(50$ ${}^{\circ }$C$)\;=\;2.497\;\times \;10^{-10}$ m${}^{2}$/s have been estimated and then plotted, in Figure 4B, with the corresponding best-fit Arrhenius function

(24) $$D_{\mathrm{eff}}\left(T\right)=D_{0}e^{-\frac{E}{R_{g}T}}\;,\;D_{0}=4.00012\times 10^{-5}m^{2}/s\;,E/R_{g}=-3872.63K.$$

The water diffusivity $D_{\mathrm{eff}}\left(T\right)$, thus estimated in the whole range of temperatures [10 ${}^{\circ }$C–50 ${}^{\circ }$C], will be used to verify the predictive capabilities of the non-isothermal model in which there are no other fitting parameters. All the other parameters have been preliminarily estimated from well-known correlations or from independent experimental measurements, like in the case of the desorption isotherms (see Appendix A).

### 4.3. The Non-Isothermal Approach

The non-isothermal model, Equations (8)–(13), rewritten for a spherical sample in terms of the water volume fraction ϕ(r,t), reads as

(25) $$\frac{\partial \phi (r,t)}{\partial t}=\frac{1}{r^{2}}\frac{\partial }{\partial r}\left(r^{2}\left(D_{\mathrm{eff}}\left(T\right)\;\frac{\partial \phi }{\partial r}-v_{s}\left(r\right)\;\phi \right)\right)\;,\;r\in \left(0,R\left(t\right)\right)$$

(26) $$\frac{\partial \left(\rho^{p}C_{p}^{p}T\left(r,t\right)\right)}{\partial t}=\frac{1}{r^{2}}\frac{\partial }{\partial r}\left(r^{2}\left(k^{p}\;\frac{\partial \phi }{\partial r}-v_{s}\left(r\right)\rho^{p}C_{p}^{p}\;T\right)\right)\;,\;r\in \left(0,R\left(t\right)\right)$$

(27) $$v_{s}\left(r\right)=D_{\mathrm{eff}}\left(T\right)\frac{\partial \phi }{\partial r}\;,\;\frac{dR(t)}{dt}=v_{s}\left(R\left(t\right)\right)=D_{\mathrm{eff}}\left(T_{b}\right)\frac{\partial \phi }{\partial r}{|}_{R(t)}$$

(28) $$\begin{array}{cc} \; & \frac{\partial \phi }{\partial r}{|}_{r=0}=0\;,\\ \; & -D_{\mathrm{eff}}\left(T_{b}\right)\frac{\partial \phi }{\partial r}{|}_{R(t)}=h_{m}\left(T_{\mathrm{av}}\right)\frac{M_{w}}{\rho_{w}}\left(\frac{p_{v}\left(T_{b}\right)}{R_{g}T_{b}}RH_{b}-\frac{p_{v}\left(T_{\infty }\right)}{R_{g}T_{\infty }}RH_{\infty }\right) \end{array}$$

(29) $$\begin{array}{cc} \; & \frac{\partial T}{\partial r}{|}_{r=0}=0\;,\\ \; & -k^{p}\frac{\partial T}{\partial r}{|}_{R(t)}=h_{T}\left(T_{\mathrm{av}}\right)\left(T_{b}-T_{\infty }\right)-\lambda_{v}\left(T_{b}\right)\rho_{w}D_{\mathrm{eff}}\left(T_{b}\right)\frac{\partial \phi }{\partial r}{|}_{R(t)} \end{array}$$

where the Relative Humidity at the air/sample interface $RH_{b}=RH{|}_{R(t)}=RH\left(\phi_{b},T_{b}\right)$ is evaluated from desorption isotherms for *Rocha* pears reported by [27] and best fitted with the Henderson model, see Appendix A. Equations (25)–(29) must be numerically integrated starting from the uniform initial conditions $\phi \left(r,0\right)=\phi_{0}≃0.9$ and $T\left(r,0\right)=T_{0}=15$ ${}^{\circ }$C. The value of $\phi_{0}$ can slightly change for different experiments because of changes in the initial total moisture content $X_{0}$ (see Table 1).

**Figure 5:**
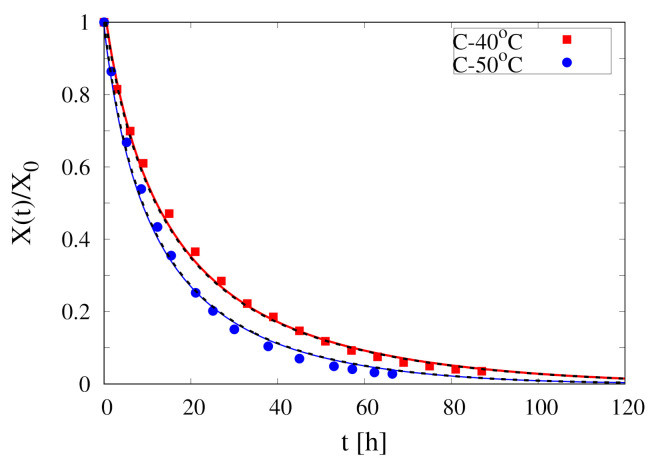
Comparison between experimental continuous dehydration curves $X\left(t\right)/X_{0}$ vs. *t* at $T_{\infty }=40$ ${}^{\circ }$C, 50 ${}^{\circ }$C and $U_{\infty }=1.28$ m/s (filled points) and model predictions (continuous and dashed lines) with D(T) given by Equation (24). Continuous red and blue curves represent the non-isothermal model Equations (25)–(29). Black dashed lines represent the non-isothermal simplified model with $T\left(r,t\right)=T_{b}\left(t\right)$, Equation (31).

Figure 5 shows the excellent agreement between experimental data, at both temperatures $T_{\infty }=40$ ${}^{\circ }$C, 50 ${}^{\circ }$C, and model predictions (continuous red and blue curves, coefficient of determination $R^{2}$ > 0.99) with no adjustable parameters. Indeed, there is no need to introduce a concentration-dependent diffusion coefficient, Equation (3), that would require the estimate of the β parameter. For this reason, we set β=0 also for all the subsequent simulations of intermittent dehydration.

The reliability of the non-isothermal model allows us to verify some hypotheses made in paragraph Section 4.1 when discussing the intrinsic limitations of the isothermal approach.

**Figure 6:**
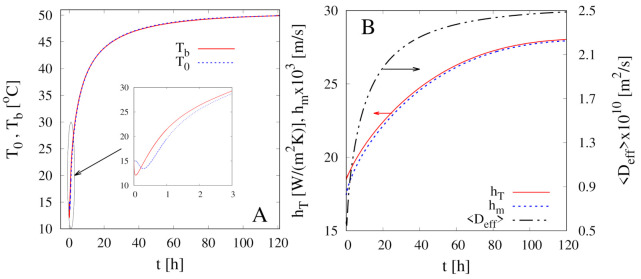
Continuous dehydration at $T_{\infty }=50$ ${}^{\circ }$C and $U_{\infty }=1.28$ m/s. (**A**) Model predictions for the temporal evolution of the temperature at the center $T_{0}\left(t\right)=T\left(0,t\right)$ and at the sample boundary $T_{b}\left(t\right)=T\left(R\left(t\right),t\right)$. (**B**) Model predictions for the temporal evolution of the heat and mass transfer coefficients $h_{T}$ and $h_{m}$, evaluated according to Equations (A10)–(A13), and of the average water effective diffusivity $<D_{\mathrm{eff}}>$, Equations (24) and (30).

Figure 6A shows the temporal evolution of the temperature at the center $T_{0}\left(t\right)=T\left(0,t\right)$ and at the sample boundary $T_{b}\left(t\right)=T\left(R\left(t\right),t\right)$ for the continuous dehydration at $T_{\infty }=50$ ${}^{\circ }$C. Due to the high value of the product thermal diffusivity $D_{T}^{p}=k^{p}/\left(\rho^{p}C_{p}^{p}\right)≃9\times 10^{-8}$ m${}^{2}$/s $\gg D_{\mathrm{eff}}$, the two temperatures $T_{0}$ and $T_{b}$ almost coincide for t>3 h. This is the only numerical result that differs from experimental observations by Silva et al. [11]. These authors observed an appreciable difference between $T_{0}$ and $T_{b}$ in the convective stage, and this is quite difficult to explain if one considers the high value of the thermal diffusivity of the pears.

As expected, both temperatures $T_{0}\left(t\right)$ and $T_{b}\left(t\right)$ require more than 40 h to get close to the asymptotic value $T_{\infty }$. This is due to the large amount of energy required for water evaporation at the air/sample interface. This effect is particularly evident in the very first hours of the dehydration process, in which both temperatures $T_{0}$ and $T_{b}$, highlighted in the inset of Figure 6, exhibit a slight decrease below the initial temperature.

Correspondingly, also the average effective water diffusivity $<D_{\mathrm{eff}}>$, shown in Figure 6B,

(30) $$<D_{\mathrm{eff}}\left(t\right)>=\frac{1}{\left(4/3\right)\pi R{\left(t\right)}^{3}}\int_{0}^{R(t)}D_{\mathrm{eff}}\left(T\left(r,t\right)\right)\;4\pi r^{2}dr$$

attains a very low value, order of $5\times 10^{-11}$ m${}^{2}$/s at the beginning of the drying process and requires more than 40 h to get close to the five times larger asymptotic value $≃2.5\times 10^{-10}$ m${}^{2}$/s.

Figure 6B also shows the temporal evolution of the heat and mass transfer coefficients $h_{T}$ and $h_{m}$, evaluated according to well-known correlations Equations (A10)–(A13) reported in Appendix A. Both $h_{T}$ and $h_{m}$ exhibit a $d^{-1/2}$ dependence on the sample diameter d(t) and therefore are increasing functions of time, mainly because of sample shrinkage.

**Figure 7:**
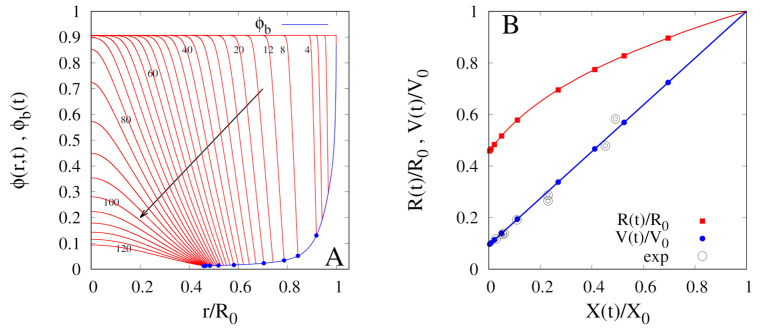
Continuous dehydration at $T_{\infty }=50$ ${}^{\circ }$C and $U_{\infty }=1.28$ m/s. (**A**) Model predictions for the temporal evolution of the water volume fraction profiles inside the shrinking sample, ϕ(r,t) vs. $r/R_{0}$. Continuous blue line highlights the water volume fraction $\phi_{b}\left(t\right)=\phi \left(R\left(t\right),t\right)$ at the solid/air interface. Blue dots indicates the values of $\phi_{b}\left(t_{i}\right)$ at increasing time instants $t_{i}=4,8,12,20,40,60,80,100,120$ h. Arrow indicates increasing values of time. (**B**) Model predictions for the temporal evolution of the rescaled sample radius $R\left(t\right)/R_{0}$ and of the rescaled sample volume $V\left(t\right)/V_{0}$ during the dehydration process. Filled points represent the same specific time instants $\left\{t_{i}\right\}$ reported in Figure (**A**). Open circles represent experimental values from [11].

The rescaled sample volume $V\left(t\right)/V_{0}$ and radius $R\left(t\right)/R_{0}$ are shown in Figure 7B as a function of the rescaled total moisture content $X\left(t\right)/X_{0}$, in agreement with experimental data (open circles, from Silva et al. [11]). Indeed, starting from these experimental shrinkage data, the hypothesis of ideal shrinkage for *Rocha* pears has been formulated and implemented in the moving-boundary model by setting $\alpha \left(\phi \right)=\alpha_{0}=1$.

The temporal evolution of the water concentration profile ϕ(r,t) is represented in Figure 7A, together with the boundary concentration $\phi_{b}\left(t\right)$ that rapidly (12 h) decreases towards the very low asymptotic value. Figure 7A clearly shows that a mixed third kind boundary condition, Equation (28), has to be applied for a correct description of all the different phases of the drying process.

A last observation regarding the temperature profiles. Figure 6A clearly shows that it is reasonable to assume that the temperature is uniform inside the sample and equal to the boundary temperature, i.e., $T\left(r,t\right)=T_{b}$. This observation allows replacing the partial differential equation Equation (26) for T(r,t) with the ordinary differential equation for the boundary temperature $T_{b}\left(t\right)$

(31) $$\begin{array}{cc} \; & \frac{d}{dt}\left(T_{b}\int_{0}^{R(t)}\left(\rho^{p}C_{p}^{p}\right)4\pi r^{2}dr\right)=\\ & \left(-h_{T}\left(T_{\mathrm{av}}\right)\left(T_{b}-T_{\infty }\right)+\lambda_{v}\left(T_{b}\right)\rho_{w}D_{\mathrm{eff}}\left(T_{b}\right)\frac{\partial \phi }{\partial r}{|}_{R(t)}\right)4\pi R^{2}\left(t\right) \end{array}$$

while the water transport equations Equations (25) and (27) remain unchanged except for the fact that $D_{\mathrm{eff}}\left(T\right)$ must be replaced with $D_{\mathrm{eff}}\left(T_{b}\right)$. The thermal inertial term $\rho^{p}C_{p}^{p}$ appears in the volume integral, right hand side of Equation (31), because it depends on ϕ(r,t) and therefore on *r* and *t* (see Appendix A).

Numerical results of the integration of this simplified model are shown in Figure 5 (black dashed lines) and are almost indistinguishable from numerical results of the more accurate non-isothermal model.

## 5. Modeling of Intermittent Drying Experiments

The non-isothermal model Equations (25)–(29) is applied to describe the intermittent drying experiments. In these experiments the air temperature $T_{\infty }$, Relative Humidity $RH_{\infty }$ and velocity $U_{\infty }$ change in time according to the cyclic repetition of three different stages, as described in Section 2 and exemplified in Figure 1. The switch between different air operating conditions is not instantaneous but it requires about 30 min [11]. This effect has been accounted for by adopting a smooth step function $\theta_{\delta }\left(t\right)$ for the switch

(32) $$\theta_{\delta }\left(t\left[h\right]\right)=\frac{1}{2}\left(1-\tanh\left(\frac{t}{\delta }\right)\right)\;,\;\delta =0.1\;h$$

shown in Figure 1. The introduction of the smooth step function also simplifies the numerical integration of the system of time-dependent partial differential equations.

**Figure 8:**
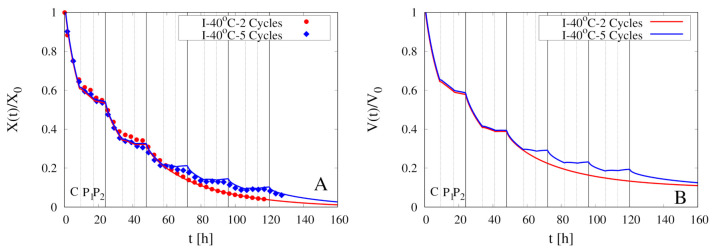
Intermittent dehydration (2 Cycles and 5 Cycles) for $T_{\infty }^{\max}=40$ ${}^{\circ }$C and $U_{\infty }^{\max}=1.28$ m/s (**A**) Comparison between experimental dehydration curves $X\left(t\right)/X_{0}$ vs. *t* (filled points) and model predictions (continuous lines) with D(T) given by Equation (24). (**B**) Model prediction of the temporal evolution for the rescaled sample volume $V\left(t\right)/V_{0}$.

Figure 8A shows the excellent agreement between experimental data for $X\left(t\right)/X_{0}$ vs. *t* and model predictions (coefficient of determination $R^{2}$ > 0.99) for two intermittent dehydration experiments at $T_{\infty }^{\max}=40$ ${}^{\circ }$C and $U_{\infty }^{\max}=1.28$ m/s. The two experiments differ in the number of cycles, 2 for the first and 5 for the second experiment, and in the initial moisture content $X_{0}$ of samples analyzed (see Table 1).

**Figure 9:**
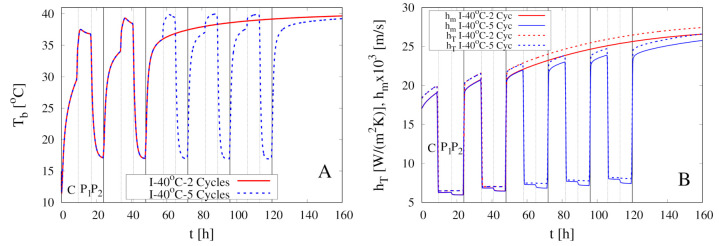
Intermittent dehydration (2 Cycles and 5 Cycles) for $T_{\infty }^{\max}=40$ ${}^{\circ }$C and $U_{\infty }^{\max}=1.28$ m/s. Model predictions for the temporal evolution of the boundary temperature $T_{b}\left(t\right)$ (**A**) and of the heat and mass transfer coefficients $h_{T}$ and $h_{m}$ (**B**).

Figure 8A clearly shows that the model is capable to perfectly describe the evolution of the total moisture content in the convective stages (C) and in the pause stages (P${}_{1}$ and P${}_{2}$). The model shows that, in agreement with experimental data, also in the pause stage $P_{1}$ (“hot-humid” pause) of the first two cycles, the sample is slightly dehydrating. Therefore, even if the air velocity is extremely low (we set $U_{\infty }=0.1$ m/s]), the sample cannot be considered “isolated” neither for the mass transfer nor for the heat transfer. The temporal evolution of the heat and mass transfer coefficients $h_{T}$ and $h_{m}$ is shown in Figure 9B.

The corresponding evolution of the rescaled sample volume $V\left(t\right)/V_{0}$ is shown in Figure 8B. It highlights how, in the last three cycles of the 5 cycles experiment when the total moisture content X(t) is low, the sample is slightly re-hydrating in the “cold-humid” pause P${}_{2}$.

The temporal evolution of the boundary temperature $T_{b}\left(t\right)$ is shown in Figure 9A. In qualitative agreement with data reported by [11] in Figure 4, $T_{b}\left(t\right)$ exhibits a rapid increase at the beginning of the hot-humid pause P${}_{1}$ due to the sudden increase of the air Relative Humidity that temporarily annihilates the heat consumption for water evaporation.

**Figure 10:**
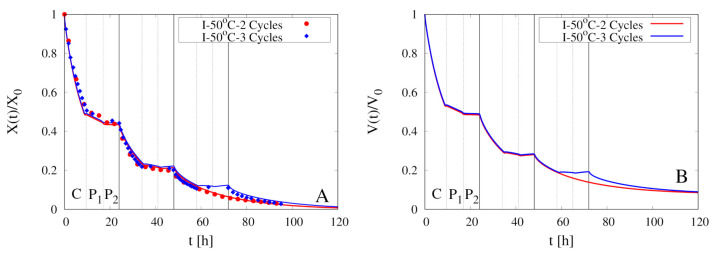
Intermittent dehydration (2 Cycles and 3 Cycles) for $T_{\infty }^{\max}=50$ ${}^{\circ }$C and $U_{\infty }^{\max}=1.28$ m/s (**A**) Comparison between experimental dehydration curves $X\left(t\right)/X_{0}$ vs. *t* (filled points) and model predictions (continuous lines) with D(T) given by Equation (24). (**B**) Model prediction of the temporal evolution for the rescaled sample volume $V\left(t\right)/V_{0}$.

Figure 10A shows the comparison between experimental data for $X\left(t\right)/X_{0}$ vs. *t* and model predictions for two intermittent dehydration experiments at the higher air temperature $T_{\infty }^{\max}=50$ ${}^{\circ }$C and the same air velocity $U_{\infty }^{\max}=1.28$ m/s. The two experiments differ in the number of cycles, 2 for the first and 3 for the second experiment.

**Figure 11:**
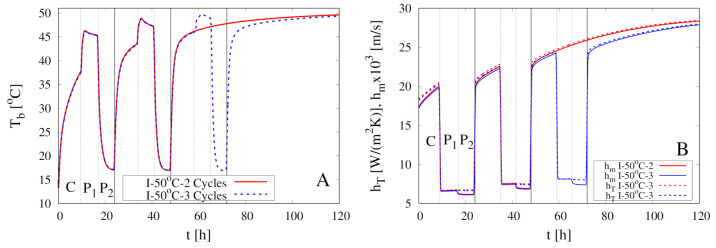
Intermittent dehydration (2 Cycles and 3 Cycles) for $T_{\infty }^{\max}=50$ ${}^{\circ }$C and $U_{\infty }^{\max}=1.28$ m/s. Model predictions for the temporal evolution of the boundary temperature $T_{b}\left(t\right)$ (**A**) and of the heat and mass transfer coefficients $h_{T}$ and $h_{m}$ (**B**).

The higher temperature implies a significantly faster dehydration which can be considered complete after about 100 h for both experiments and in perfect agreement with experimental dehydration curves. Model predictions for the temporal evolution of the rescaled sample volume $V\left(t\right)/V_{0}$, the boundary temperature $T_{b}\left(t\right)$ and the heat and mass transfer coefficients $h_{T}$ and $h_{m}$ are shown in Figure 10B and Figure 11A,B, respectively. A qualitative behavior, similar to that obtained for intermittent experiments at the lower temperature $T_{\infty }^{\max}=40$ ${}^{\circ }$C, can be observed.

**Figure 12:**
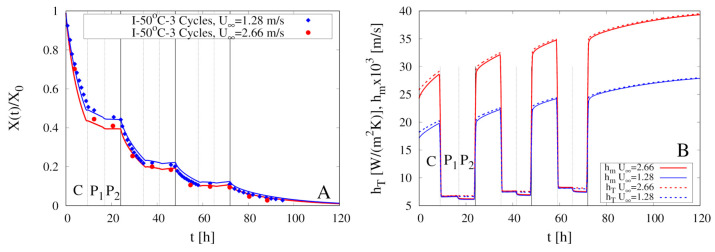
Intermittent dehydration (3 Cycles) for $T_{\infty }^{\max}=50$ ${}^{\circ }$C and two different air velocities $U_{\infty }^{\max}=1.28$ m/s and 2.66 m/s. (**A**) Comparison between experimental dehydration curves $X\left(t\right)/X_{0}$ vs. *t* (filled points) and model predictions (continuous lines) with D(T) given by Equation (24). (**B**) Model predictions for the temporal evolution of the heat and mass transfer coefficients $h_{T}$ and $h_{m}$.

The effect of the air velocity $U_{\infty }$ on the dehydration process is also investigated, as a further check of the validity of the correlation functions adopted for the estimate of the heat and mass transfer coefficients $h_{T}$ and $h_{m}$. Figure 12A compares experimental results and model predictions for the 3 Cycles intermittent experiments at $T_{\infty }^{\max}=50$ ${}^{\circ }$C characterized by two different air velocities $U_{\infty }^{\max}=1.28$ m/s and 2.66 m/s.

Experimental results for $X\left(t\right)/X_{0}$, shown in Figure 12A, highlight a slight effect of $U_{\infty }^{\max}$ on the dehydration curves, mainly in the first two cycles of the dehydration process, while the final dehydration time is substantially unaffected by $U_{\infty }^{\max}$. Model predictions, in excellent agreement with experimental data, show that, more than doubling the air speed $U_{\infty }^{\max}$, the heat and mass transfer coefficients increase by a factor of about 1.5 (see Figure 12B), and this affects the dehydration rate mainly when the sample moisture content is still high.

Model predictions confirm the experimental findings by [11]. A significant reduction of the final dehydration time can be achieved by increasing the operating temperature from 40 ${}^{\circ }$C to 50 ${}^{\circ }$C while an increase in air speed has proven ineffective.

**Figure 13:**
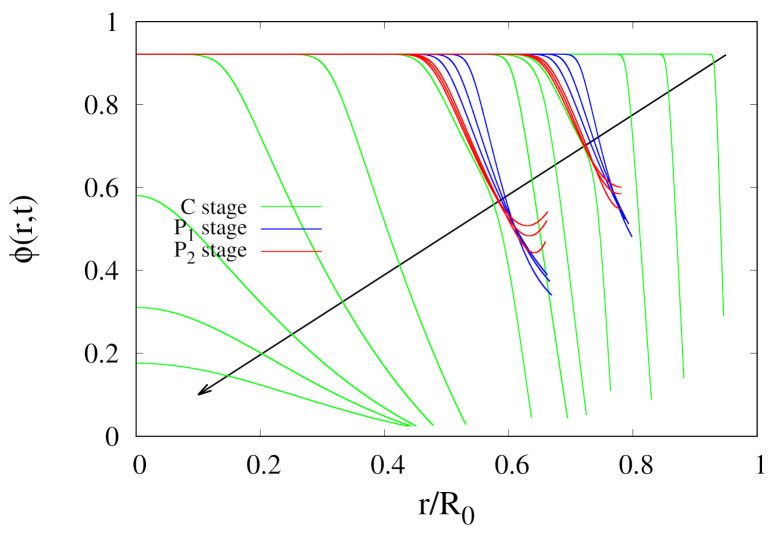
Temporal evolution of water volume fraction profiles ϕ(r,t) for the intermittent dehydration experiment (2 Cycles) at $T_{\infty }^{\max}=50$ ${}^{\circ }$C and $U_{\infty }^{\max}=1.28$ m/s. Arrow indicates increasing time instants $t_{i}=\left(2,5,8\right),\left(12,14,16\right),\left(19,21,23\right)$, (25,28,31),(36,38,40),(43,45,47),(50,70,90,110,120,130) h. Green, blue and red curves highlight concentration profiles in the Convective (C), hot humid (P${}_{1}$) and cold humid (P${}_{2}$) stages, respectively.

A final remark must be made on the influence of the pause stages on moisture gradients inside the sample. Quite often, intermittent drying has to be preferred to continuous drying because, during each tempering period, a redistribution of internal moisture within the drying material occurs. A reduction in moisture gradients [8] decreases the probability of concentration-induced stress and fissure. This occurs, for example, for rice grains [30] and other seeds, i.e. for food with very small dimensions, order of millimeters. In the present case of whole pears, the effect of moisture homogenization in the pause stages is extremely small as shown in Figure 13. Red and blue curves in Figure 13 represent the temporal evolution of the water volume fraction profiles ϕ(r,t) in the two pause stages P${}_{1}$ and P${}_{2}$ in the intermittent experiment with two cycles at $T_{\infty }^{\max}=50$ ${}^{\circ }$C and $U_{\infty }^{\max}=1.28$ m/s. No significant gradient reduction is observed in the pause stages.. This effect is intrinsically due to the larger sample dimension, about 5 cm, and to the low effective diffusivity $D_{\mathrm{eff}}$.

## 6. Conclusions

This article presents the non-isothermal formulation of the moving-boundary model for food dehydration, recently proposed by [13,14], in which sample shrinkage is accounted for via the introduction of the pointwise shrinkage velocity that depends on the local volumetric water flux. A convection-diffusion heat transport equation, affected by sample shrinkage, heat transfer and water evaporation at the sample surface, is added to the convection-diffusion transport equation for water concentration.

The non-isothermal model is successfully applied to experimental data of continuous and intermittent drying of *Rocha* pears reported by Silva and coworkers.

No particular analytical/computational efforts were required to estimate the shrinkage proportionality factor α(ϕ) because pears exhibit a net *ideal* shrinkage and a constant value $\alpha \left(\phi \right)=\alpha_{0}=1$ can be assumed a priori.

The excellent predictive capability of the non-isothermal model makes it a useful tool for optimizing intermittent dehydration procedures at a laboratory and industrial level.

From the strictly theoretical point of view, the model showed that it is not necessary, if not wrong, to introduce a time-dependent water diffusivity. On the contrary, it is necessary (1) to take into account the dependence of diffusivity on temperature and therefore (2) to follow the temporal evolution of the temperature, at least the surface temperature.

## Figures and Tables

**Table 1:** Initial total moisture content $X_{0}$ [kg water/kg dry solid] and sample dimension $d_{0}$ [cm] for different continuous (C) and intermittent (I) drying experiments. * Dehydration curves shown in Figure 2. ** Dehydration curve shown in Figure 12A.

Experiment	Type	Cycles	$T_{\infty }$	$U_{\infty }$	$X_{0}$	$d_{0}$
			[${}^{\circ }$C]	[m/s]		[cm]
C-40 ${}^{\circ }$C *	C	-	40	1.28	5.64	5.30
I-40 ${}^{\circ }$C-2 Cycles *	I	2	40	1.28	6.48	5.36
I-40 ${}^{\circ }$C-5 Cycles *	I	5	40	1.28	5.37	5.30
C-50 ${}^{\circ }$C *	C	-	50	1.28	5.55	5.24
I-50 ${}^{\circ }$C-2 Cycles *	I	2	50	1.28	6.21	5.32
I-50 ${}^{\circ }$C-3 Cycles I *	I	3	50	1.28	6.25	5.69
I-50 ${}^{\circ }$C-3 Cycles II **	I	3	50	2.66	7.24	5.43

**Figure A1:**
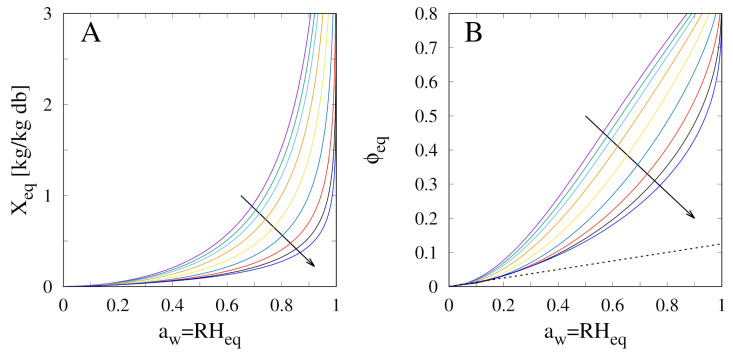
Desorption isotherms for different temperatures $T_{\infty }$ in the range [10 ${}^{\circ }$C–50 ${}^{\circ }$C]. Arrows indicate increasing values of the temperature $T_{\infty }$. (**A**) $X_{\mathrm{eq}}$ vs. water activity $a_{w}$. (**B**) $\phi_{\mathrm{eq}}$ vs. $a_{w}$. The dashed line indicates the approximated linear behaviour Equation (A3).

## Nomenclature

Symbols		
$a_{w}$	[-]	Water activity
$Bi_{\infty }$	[-]	Asymptotic Biot number, Equation (A16)
$c_{w}$	[g water/m${}^{3}$ product]	Water mass concentration
$C_{p}^{p}$,$C_{p}^{s}$,$C_{p}^{w}$	[J/(g K)]	Product, solid and water specific heat capacity
$k^{p}$,$k^{s}$,$k^{w}$	[W/(m K)]	Product, solid and water thermal conductivity
*d*	[m]	Sample diameter
$D_{\mathrm{eff}}$	[m${}^{2}$/s]	Effective water diffusivity
$D_{v}^{\mathrm{air}}$	[m${}^{2}$/s]	Vapor in air diffusivity
$D_{T}^{\mathrm{air}}$	[m${}^{2}$/s]	Air thermal diffusivity
$D_{T}^{p}$	[m${}^{2}$/s]	Product thermal diffusivity
$h_{m}$	[m/s]	Mass transfer coefficient
$h_{T}$	[W/(m${}^{2}$ K)]	Heat transfer coefficient
*J*	[h${}^{-1}$]	Dehydration rate
$J_{d}$	[g water/(s m${}^{2}$)]	Water diffusive mass flux
$k^{\mathrm{air}}$	[W/(m K)]	Air thermal conductivity
$K_{eq}$	[-]	Equilibrium constant, Equation (A3)
Nu	[-]	Nusselt number
*p*	[Pa]	Vapor partial pressure
$p_{v}$	[Pa]	Saturated vapor pressure
Pr	[-]	Prandl number
*r*	[m]	Radial coordinate
*R*	[m]	Sample radius
$R_{g}$	[J/(mol K)]	Gas constant
Re	[-]	Reynolds number
RH	[-]	Relative humidity
Sc	[-]	Schmidt number
Sh	[-]	Sherwood
*t*	[s]	Time
*T*	[K]	Temperature
$T_{\mathrm{av}}$	[K]	Average film temperature
*V*	[m${}^{3}$]	Sample volume
$v_{s}$	[m/s]	Shrinkage velocity
$x^{w}$	[-]	Water weight fraction
*X*	[kg water/kg dry solid]	Total moisture content

Greek Symbols		
α	[-]	Shrinkage factor
$\gamma_{0}$	[-]	smallest positive root of Equation (A18)
$\lambda_{v}$	[J/g]	Heat of water vaporization
$\nu^{air}$	[m/s]	Air kinematic viscosity
$\rho^{p}$	[g product/cm${}^{3}$ product]	Product density
$\rho^{s}$	[g solid/cm${}^{3}$ solid]	Solid (pulp) density
$\rho^{w}$	[g water/cm${}^{3}$ water]	Water density
ϕ	[-]	water volume fraction
Subscripts		
0	Initial	
*b*	Sample surface (boundary)	
eq	Equilibrium	
*∞*	Asymptotic or at infinite distance	

## Appendix A

The Relative Humidity at the air/sample interface is evaluated from desorption isotherms for *Rocha* pears reported by [27] and best fitted with the Henderson model

(A1) $$RH_{eq}\left(X_{eq},T\right)=1-\exp\left(-a\left(T\right)\;TX_{eq}^{b(T)}\right)\;,\;T\left[K\right]\;,\;X_{eq}\left[\mathrm{kg}\;\text{w}/\mathrm{kg}\;\mathrm{dry}\;\mathrm{solid}\right]$$

The Henderson model exhibits an explicit dependence on the desorption temperature *T* and is capable to accurately describe the influence of the temperature on desorption curves in the range [20 ${}^{\circ }$C–40 ${}^{\circ }$C]. The values of the two parameters a(T) and b(T), entering the desorption isotherm model Equation (A1) are a=0.0049,0.0062,0.0092 and b=0.5739, 0.5754, 0.6449 for T=20,30,40 ${}^{\circ }$C, respectively [27]. In order to estimate the desorption isotherm in the whole range of temperature [10 ${}^{\circ }$C–50 ${}^{\circ }$C] the two parameters a(T) and b(T) have been estimated, from the values listed above, by piecewise cubic interpolation. The resulting isotherms, in the whole range [10 ${}^{\circ }$C–50 ${}^{\circ }$C], are plotted in Figure A1A,B as $X_{eq}$ vs. $a_{w}$ (Figure A1A) and as $\phi_{eq}$ vs. $a_{w}$ (Figure A1B). The following equation

(A2) $$\phi_{eq}=\frac{\rho^{s}X_{eq}}{X_{eq}\rho^{s}+\rho^{w}}$$

relating the water volume fraction $\phi_{eq}$ to the moisture content $X_{eq}$ has been adopted.

From Figure A1B, it can be observed that the desorption isotherm, for high temperatures T∈[40 ${}^{\circ }$C–50 ${}^{\circ }$C] and small values of $a_{w}<0.2$ can be readily approximated with a linear function (dashed line)

(A3) $$\phi_{eq}=a_{w}/K_{eq}\;,\;K_{eq}≃8$$

The density of the solid (pulp) $\rho^{s}=1.73$ [g solid/cm${}^{3}$ solid] is estimated from the initial product (pear) density $\rho_{0}^{p}≃1.07$ [g product/cm${}^{3}$ product] and from the initial moisture content $X_{0}≃5.25$ [kg water/kg dry solid], reported by [31] for *Rocha* pears, as follows

(A4) $$\rho^{s}=\frac{\rho_{0}^{p}\rho^{w}}{\rho^{w}\left(1+X_{0}\right)-\rho_{0}^{p}X_{0}}$$

The pear density $\rho^{p}$ is evaluated as the average of the water and solid (pulp) densities, averaged with respect to their volume fractions

(A5) $$\rho^{p}=\rho^{w}\phi +\rho^{s}\left(1-\phi \right)$$

The pear specific heat capacity $C_{p}^{p}$ [J/(g K)] is evaluated as the average of the water and solid specific heat capacities, averaged with respect to their weight fractions

(A6) $$C_{p}^{p}=C_{p}^{w}x_{w}+C_{p}^{s}\left(1-x_{w}\right)\;,\;x_{w}=\phi \;\left(\rho^{w}/\rho^{p}\right)$$

The pear thermal conductivity $k^{p}$ [W/(m K)] is evaluated from a parallel model

(A7) $$\frac{1}{k^{p}}=\frac{\phi }{k^{w}}+\frac{1-\phi }{k^{s}}$$

where $k^{w}$ and $k^{s}$ are the water and solid thermal conductivities, respectively.

Since pears contain mainly water and carbohydrate [32], the thermal conductivity $k^{s}$ and the specific heat capacity $C_{p}^{s}$ of the solid phase are estimated from that of carbohydrate [33]

(A8) $$\begin{array}{ccc} k^{s}\left[W/(m\;K)\right] & = & 2.01\times 10^{-1}+1.39\times 10^{-3}-4.33\times 10^{-6}T^{2}\;,\;T{[}^{\circ }C] \end{array}$$

(A9) $$\begin{array}{ccc} C_{p}^{s}\left[J/(g\;K)\right] & = & 1.5488+1.9625\times 10^{-3}T-5.9399\times 10^{-6}T^{2}\;,\;T{[}^{\circ }C] \end{array}$$

The heat and mass transfer coefficients $h_{T}$ [W/(m K)] and $h_{m}$ [m/s]

(A10) $$\begin{array}{c} h_{T}\left(T_{av},d\right)=\frac{Nu\left(T_{av},d\right)k^{\mathrm{air}}\left(T_{av}\right)}{d}\;,\\ h_{m}\left(T_{av},d\right)=\frac{Sh\left(T_{av},d\right)D_{v}^{\mathrm{air}}\left(T_{av}\right)}{d} \end{array}$$

are evaluated from the well known correlation functions for the Nusselt Nu and Sherwood Sh numbers around a sphere [34]

(A11) $$\begin{array}{ccc} Nu(T_{av},d) & = & 2+0.6Re^{1/2}Pr^{1/3}=\\ & & 2+{\left(\frac{U_{\infty }d}{\nu_{\mathrm{air}}\left(T_{av}\right)}\right)}^{1/2}{\left(\frac{\nu_{\mathrm{air}}\left(T_{av}\right)}{D_{T}^{\mathrm{air}}\left(T_{av}\right)}\right)}^{1/3} \end{array}$$

(A12) $$\begin{array}{ccc} Sh(T_{av},d) & = & 2+0.6Re^{1/2}Sc^{1/3}=\\ & & 2+{\left(\frac{U_{\infty }d}{\nu_{\mathrm{air}}\left(T_{av}\right)}\right)}^{1/2}{\left(\frac{\nu_{\mathrm{air}}\left(T_{av}\right)}{D_{v}^{\mathrm{air}}\left(T_{av}\right)}\right)}^{1/3}\;. \end{array}$$

Both $h_{T}$ and $h_{m}$ change in time because all the physical properties of the humid air are evaluated at the film mean temperature $T_{av}\left(t\right)=\left(T_{b}\left(t\right)+T_{\infty }\right)/2$ which changes in time. Moreover, also the sample diameter d(t) and the Reynolds number are changing during the course of the dehydration process.

The thermo-physical parameters of moist air (density, viscosity, thermal conductivity and thermal diffusivity) are evaluated as that for dry air since, for T≤50 ${}^{\circ }$C, Relative Humidity has a small influence on thermo-physical properties of moist air [35,36,37].

## Appendix B

Let us focus on a continuous dehydration process in which the air properties $T_{\infty }$, $RH_{\infty }$ and $U_{\infty }$ are set to fixed and constant values. On long time scales, when the water volume fraction ϕ is low everywhere inside the sample, it can be reasonably assumed that

1. the sample temperature has already reached its asymptotic value $T\left(r,t\right)=T_{\infty }$
2. the sample radius can be approximated with its asymptotic value $R_{\infty }$
  (A13) $$\frac{R_{\infty }}{R_{0}}={\left(\frac{V_{\infty }}{V_{0}}\right)}^{1/3}$$
3. the convective-shrinkage contribution $D_{\mathrm{eff}}\frac{\partial \phi }{\partial r}\phi$ is negligible compared to the diffusive term $-D_{\mathrm{eff}}\frac{\partial \phi }{\partial r}$, and a purely diffusive transport equation can be adopted
  (A14) $$\frac{\partial \phi (r,t)}{\partial t}=D_{\mathrm{eff}}\left(T_{\infty }\right)\frac{1}{r^{2}}\frac{\partial }{\partial r}\left(r^{2}\frac{\partial \phi }{\partial r}\right)\;,\;r\in \left(0,R_{\infty }\right)$$
4. the nonlinear desorption isotherm at $T=T_{\infty }$ can be approximated with a linear behaviour valid for small RH, Equation (A3), shown in Figure A1B (black dashed line). Therefore, Equation (A14) can be solved with the simplified boundary conditions
  (A15) $$\frac{\partial \phi }{\partial r}{|}_{r=0}=0\;,\;-R_{\infty }\frac{\partial \phi }{\partial r}{|}_{R_{\infty }}=Bi_{\infty }\left(\phi_{b}-\phi_{\infty }\right)$$
  where $\phi_{b}=RH_{b}/K_{\mathrm{eq}}$, $\phi_{\infty }=RH_{\infty }/K_{\mathrm{eq}}$ and $Bi_{\infty }$ represents the asymptotic mass Biot number
  (A16) $$Bi_{\infty }=\frac{h_{m}\left(T_{\infty }\right)R_{\infty }}{D_{\mathrm{eff}}\left(T_{\infty }\right)}\frac{M_{w}}{\rho_{w}}\frac{p_{v}\left(T_{\infty }\right)}{R_{g}T_{\infty }}K_{\mathrm{eq}}\left(T_{\infty }\right)$$

The asymptotic solution of Equations (A14) and (A15), written in terms of the moisture ratio $X_{r}\left(t\right)$, reads as [22,38]

(A17) $$X_{r}\left(t\right)=\frac{X\left(t\right)-X_{eq}}{X_{0}-X_{eq}}∼\exp\left[-\gamma_{0}^{2}\frac{tD_{\mathrm{eff}}\left(T_{\infty }\right)}{R_{\infty }^{2}}\right]\;\mathrm{valid}\;\mathrm{for}\;\mathrm{large}\;t\;$$

where $\gamma_{0}$ is the smallest positive root of the equation

(A18) $$\gamma \cot(\gamma )+Bi_{\infty }-1=0.$$

It is straightforward to verify that the asymptotic exponential behaviour Equations (A17) implies a linear behaviour for the dehydration rate *J* vs. $X_{r}$ for small values of $X_{r}$

(A19) $$J=-\frac{dX_{r}}{dt}=D_{\mathrm{eff}}\left(T_{\infty }\right)\;\frac{\gamma_{0}^{2}}{R_{\infty }^{2}}X_{r}\;\mathrm{valid}\;\mathrm{for}\;\mathrm{small}\;X_{r}$$

Equation (A19) can be used to estimate the effective water diffusivity $D_{\mathrm{eff}}$ at $T=T_{\infty }$ from the initial linear scaling of the experimental dehydration-rate curve $J=\theta \left(T_{\infty }\right)X_{r}$. The dehydration-rate curves are shown in Figure 4A for the two continuous dehydration experiments at $T_{\infty }=40$ ${}^{\circ }$C and 50 ${}^{\circ }$C.

The estimate of $D_{\mathrm{eff}}\left(T_{\infty }\right)$ from Equation (A19) is not so straightforward as it requires the solution of a nonlinear equation for $D_{\mathrm{eff}}\left(T_{\infty }\right)$ because $\gamma_{0}$ is a nonlinear function of $Bi_{\infty }$ and $Bi_{\infty }$ depends on $D_{\mathrm{eff}}\left(T_{\infty }\right)$, Equation (A16).

This estimate strongly simplifies in the case of high values of the Biot number $Bi_{\infty }$ because $\gamma_{0}$ is an increasing function of $Bi_{\infty }$, saturating towards π for $Bi_{\infty }\to \infty$. For $Bi_{\infty }>10^{2}$, $\gamma_{0}$ can be well approximated as $\gamma_{0}≃\pi$. In the present case, for an effective diffusivity $D_{\mathrm{eff}}≃10^{-10}$ m${}^{2}$/s, the Biot number is $Bi_{\infty }>10^{3}>>10^{2}$ for both temperatures $T_{\infty }=40$ ${}^{\circ }$C and 50 ${}^{\circ }$C. Consequently, no iterative procedure is required and the effective diffusivity $D_{\mathrm{eff}}\left(T_{\infty }\right)$ can be directly estimated from the initial slope $\theta (T_{\infty })$ of the corresponding experimental dehydration-rate curves as follows

(A20) $$J=\theta \left(T_{\infty }\right)X_{r}=D_{\mathrm{eff}}\left(T_{\infty }\right)\;\frac{\pi^{2}}{R_{\infty }^{2}}X_{r}=D_{\mathrm{eff}}\left(T_{\infty }\right)\;\frac{\pi^{2}}{R_{0}^{2}}{\left(\frac{V_{\infty }}{V_{0}}\right)}^{-\frac{2}{3}}X_{r}.$$

where the only unknown quantity is $D_{\mathrm{eff}}\left(T_{\infty }\right)$.

If we are assuming a concentration dependent water diffusivity, in agreement with Equation (3), Equation (A20) permits us to estimate solely the asymptotic value $D_{\infty }$. No information on the β value can be obtained from the asymptotic analysis. If we assume a concentration independent water diffusivity, then $D_{\mathrm{eff}}$ and $D_{\infty }$ coincide.
